# Supplementary material for: Chromothripsis during telomere crisis is independent of NHEJ, and consistent with a replicative origin
Source: Genome Res. 2019 May;29(5):737–49. doi: 10.1101/gr.240705.118 (PMC6499312; doi:10.1101/gr.240705.118)
Supplement: Supplemental Material [file supp_gr.240705.118_Supplemental_file_1.zip › contigs/annotated_contigs/DB46/contig.5.DB46_length_419_mean_cov_5.03102625298.docx]

**DB46_length_419_mean_cov_5.03102625298**

```
CCGATCT|AGGGTGGCTGTGAGGATTAAATGCATTTGTACACGCCTGTAATCCCAGCACTTTGGGAGGCCGAGGCAGGCAGATC|ACTT
        >chr3:149799477-149799566 + E=3e-31 p=0e+00                                 >ch
GAGGTCAGGAG|TTTGAGACCAGCCTGGCCAACATGGTGAAACCCTGCCTTTACTAAAAATACAAAAATTAGCTGGGCTTGGTTGCAGG
r20:23244854-23244964 - E=1e-33 p=0e+00
CACCTGTAATCCCAGCTG|AAAATACAAAAATTAGCTGGGCTTGGTTGCAGGCACCTGTAATCCCAGCTGCTCGGGAGGCTGAAGCAGA
                    >chr8:38025947-38026032 + E=1e-27
AGAATCACTTGAACC|CTGGGACACAGA|TTGCAGTGAGCCCAGATTGCGCTATTGCACTCCAGCCTGGGTGACAGTG|ACA|GAACAT
                            >chr10:121629895-121629944 + E=6e-12                >chr5
CCTAAATGCCTTAACTTAATCATTCCGCCTTCTATGCATGTAACAAAATATCATATGTACCCAATAAATAT:3233050-3233127 - E=8e-35
```
